# Influenza vaccination strategies for 2020-21 in the context of COVID-19

**DOI:** 10.7189/jogh.10.021102

**Published:** 2020-12

**Authors:** Xin Wang, Durga Kulkarni, Marshall Dozier, Karen Hartnup, John Paget, Harry Campbell, Harish Nair, for the Usher Network for COVID-19 Evidence Reviews (UNCOVER) group

**Affiliations:** 1 Centre for Global Health, Usher Institute, University of Edinburgh, UK; 2 Information Services, University of Edinburgh, Edinburgh, UK; 3 Netherlands Institute for Health Services Research, Utrecht, the Netherlands

## Abstract

**Background:**

Influenza vaccination prevents people from influenza-related diseases and thereby mitigates the burden on national health systems when COVID-19 circulates and public health measures controlling respiratory viral infections are relaxed. However, it is challenging to maintain influenza vaccine services as the COVID-19 pandemic has the potential to disrupt vaccination programmes in many countries during the 2020/21 winter. We summarise available recommendations and strategies on influenza vaccination, specifically the changes in the context of the COVID-19 pandemic.

**Methods:**

We searched websites and databases of national and international public health agencies (focusing on Europe, North and South America, Australia, New Zealand, and South Africa). We also contacted key influenza immunization focal points and experts in respective countries and organizations including WHO and ECDC.

**Results:**

Available global and regional guidance emphasises the control of COVID-19 infection in immunisation settings by implementing multiple measures, such as physical distancing, hand hygiene practice, appropriate use of personal protective equipment by health care workers and establishing separate vaccination sessions for medically vulnerable people. The guidance also emphasises using alternative models or settings (eg, outdoor areas and pharmacies) for vaccine delivery, communication strategies and developing registry and catch-up programmes to achieve high coverage. Several novel national strategies have been adopted, such as combining influenza vaccination with other medical visits and setting up outdoor and drive through vaccination clinics. Several Southern Hemisphere countries have increased influenza vaccine coverage substantially for the 2020 influenza season. Most of the countries included in our review have planned a universal or near universal influenza vaccination for health care workers, or have made influenza vaccination for health care workers mandatory. Australia has requested that all workers and visitors in long term care facilities receive influenza vaccine. The UK has planned to expand the influenza programme to provide free influenza vaccine for the first time to all adults 50-64 years of age, people on the shielded patient list and their household members and children in the first year of secondary school. South Africa has additionally prioritised people with hypertension for influenza vaccination.

**Conclusions:**

This review of influenza vaccination guidance and strategies should support strategy development on influenza vaccination in the context of COVID-19.

Influenza virus causes epidemics each year which usually occur in winter and spring in temperate regions and throughout the year (with more than one peak) in tropical regions [1,2]. Influenza virus can cause mild to severe disease in the human population and accounts for about 291 000-646 000 respiratory deaths annually, placing a substantial burden on health care systems globally [3]. Influenza activity in the past few months was lower than expected in many countries due to the implementation of lockdown and physical distancing measures which were adopted to minimise the transmission of severe acute respiratory syndrome coronavirus 2 (SARS-CoV-2) that causes coronavirus disease (COVID-19) [4-7]. However, it is expected that influenza activity will increase in the upcoming (2020-21) season as these measures are being lifted, and there are concerns that national health systems could be overwhelmed by the co-circulation of COVID-19 and influenza viruses [8]. Additionally, influenza virus is the most frequent secondary pathogen identified in patients with COVID-19, due to paucity of data and conflicting results in published literature regarding the outcome of such co-infections, the clinical significance of such co-infections remains unclear [9-13]. For example, a large case-control study in England, UK found that influenza virus co-infection was associated with increased COVID-19 severity and higher risk of mortality, while another case-control study among hospitalised COVID-19 patients in Wuhan, China reported an opposite impact of influenza co-infection [11,13]. Whilst a COVID-19 vaccine does not yet exist, influenza vaccination, although does not provide 100% protection, protects many people, especially medically vulnerable people, from influenza-related severe morbidity and mortality and thereby reduces the burden on national health systems in the upcoming influenza season [14]. However, influenza vaccination services can be difficult to sustain in the presence of COVID-19 [15,16]. In this review, we provide a summary of regional and national recommendations and strategies on influenza vaccination, specifically the changes in the context of COVID-19.

## METHODS

We conducted a rapid review on regional and national influenza vaccination strategies and generic guidance on immunization in the context of the COVID-19 pandemic. We searched websites and databases of national and international public health agencies (focusing on Europe, North and South America, Australia, New Zealand, and South Africa) for relevant documents and press releases. We also contacted key influenza immunization focal points and experts in respective countries and organizations including World Health Organisation (WHO), the WHO Regional Office for Americas (PAHO) and European Centre for Disease Prevention and Control (ECDC).

## RESULTS

**Table 1** provides a summary of results by region and country. Globally, there is generic guidance on immunization during the COVID-19 pandemic published by WHO, although not specific to influenza. WHO recommends that immunisation should be prioritised and continued during the COVID-19 pandemic and, where suspended, countries should reinstate immunisation services as soon as possible [15]. They recommend that countries should suspend mass vaccination campaigns temporarily and focus on the provision of vaccines from fixed site immunisation services, using additional and / or alternative locations to increase vaccine coverage. They also recommend that countries develop effective communication strategies and catch-up vaccination programmes to achieve a high vaccine coverage. On the basis of this guiding principle, PAHO specifically recommends that influenza vaccination should be prioritized whilst the capacity of the health system is intact and the provision of essential health services continues [19]. To limit the spread of COVID-19 in immunisation settings, PAHO specifically recommends conducting vaccination sessions in well-ventilated areas with frequent environmental disinfection, scheduling vaccination appointments to avoid crowding, implementing hand hygiene practice and appropriate use of personal protective equipment by health care workers. To protect medically vulnerable people, PAHO recommends establishing separate vaccination sessions exclusively for older people and people with pre-existing medical conditions. It is recommended that people with confirmed or suspected COVID-19 outside health care facilities postpone vaccination until symptoms resolve following negative test results of COVID-19 or 14 days later if testing is not feasible. People with COVID-19 in health care facilities should be vaccinated upon recovery and prior to discharge. Staff must not attend vaccination if they are unwell. European Commission includes influenza vaccination as one of the key strategies for “EU health preparedness for COVID-19 outbreaks” this winter (van der Hoek W 2020, personal communication, 4 September).

**Table 1:** Summary of changes in influenza immunisation recommendations during the COVID-19 era

Country or region	Adjustment in the influenza vaccination priority group list	Approaches to increase influenza vaccine coverage	Infection control measures against SARS-CoV-2 transmission in immunisation service settings	Others
WHO [15,17,18]	1. No change in the priority groups. Influenza vaccination of health workers, older adults, and pregnant women is advised. 2. Recommend providing annual influenza vaccination and pneumococcal conjugate vaccines to employees and staff in long-term care facilities, according to local policies.	Guiding principles: 1. Immunization is a core health service that should be prioritized and safeguarded for continuity during the COVID-19 pandemic. 2. If immunization services must be diminished or suspended, countries should reinstate and reinvigorate immunization services as soon as possible. 3. Recommend using subnational data to drive improvements in vaccination coverage. 4. Countries will need to develop catch-up vaccination strategies if the provision of immunization services are negatively impacted by COVID-19. 5. Countries should implement effective communication strategies to address safety concerns of the community and re-establish community demand for vaccination.	Guiding principles: 1. Immunization delivery strategies may need to be adapted to ensure the safety of health workers, caregivers and the community. Recommend tailoring local immunization programmes to address local challenges, improving the design of clinics, and accelerating the integration of immunization campaigns. 2. Mass vaccination campaigns should be temporarily suspended. Countries should monitor and re-evaluate the necessity for delaying mass vaccination campaigns regularly. The decision to conduct outbreak response mass vaccination campaigns will require a careful risk-benefit analysis on a case-by-case basis. 3. Where health system capacity is intact and essential health services are operational (eg, adequate human resources, adequate vaccine supply), fixed site immunization services should be executed while maintaining physical distancing measures and appropriate infection control precautions. 4. Alternative strategies (eg, outreach or mobile services) should be implemented to ensure the safety of the health workers and community and to optimize service delivery in the local context.	
WHO Region of the Americas [19]; Latin American Countries (Peru, Uruguay, Ecuador, Chile) [20-24]	1. No change in target groups but have assigned specific dates /appointments for specific age groups and prioritised at-risk population. 2. Costa Rica prioritized at risk-groups and assigned vaccination dates by last name, with adults older than 65 y having priority from 7:30 to 10:00 am 3. Argentina prioritised health care workers and adults older than 65 y.	Guidance in WHO Region of the Americas: 1. Influenza vaccination should be prioritised when the capacity of the health system is intact and the provision of essential health services continues. 2. Implement a personalized communication strategy to promote the continued use of immunization services.3. Use registry systems to record and follow up vaccination. At country level: 4. Chile has achieved a high coverage of over 98% in target population (translating to seven million additional doses compared to 2019-20). 5. Argentina recommends that the time for termination of influenza vaccination campaign be decided on the prevailing epidemiological situation.	Guidance at the regional and country level: 1. Mass vaccination campaigns should be temporarily suspended. Countries should intensify vaccination as soon as possible, once health services go back to normal. 2. Conduct vaccination sessions in well-ventilated areas that are frequently disinfected. Establish exclusive vaccination sessions for older people and people with pre-existing medical conditions. 3. Hand hygiene practice. 4. Only one family member is allowed to accompany the vaccinee. 5. Scheduled vaccination appointments. 6. Using other essential preventive health services and outdoor spaces to provide vaccination. 7. Separate vaccination posts from other posts. 8. Appropriate use of medical/surgical masks by immunization personnel. Vaccinators should comply with guidelines on clothing. 9. For those with suspected or confirmed COVID-19 outside health care facilities, vaccination is only conducted when individual is free of COVID-19 symptoms, preferably following two consecutive tests negative for COVID-19 (conducted 24 h apart) or deferring the vaccination for 14 d after system resolution. If a person with confirmed or suspected COVID - 19 is in health care facilities, this person should be vaccinated upon recovery and prior to discharge. 10. Vaccinators must not attend work if they are unwell.	1. Identify people with suspected COVID-19 and refer them for medical consultation. 2. The cold chain storage capacity may need to be expanded.
Canada [25,26]	No change in the priority groups: all pregnant women, adults and children with chronic health conditions, adults older than 65 y, children 6-59 mo, indigenous peoples, residents of nursing homes and other chronic care facilities, health care workers and other care providers in facilities and community settings, and contacts (both adults and children) of individuals at high-risk irrespective of whether the individual at high risk has been vaccinated. Healthcare workers and other care providers in facilities and community settings are urged to get influenza vaccine.		1. Consider alternate models of influenza vaccine delivery. 2. Implement multiple measures to maintain physical distancing (two meters): scheduled appointments, using signage and floor markings, spacing chairs, installing barriers, monitoring entries, exits, waiting areas and the queue. 3. People will be screened before entry for COVID-19 symptoms. Those with symptoms should be instructed to perform hand hygiene, put on a medical mask and be redirected for assessment. Influenza vaccination should usually be postponed in people with serious acute illnesses until their symptoms have abated. 4. Providing hand sanitizer throughout the venue. 5. Ensure frequent environmental disinfection. 6. Appropriate use of personal protective equipment by all staff. 7. Appropriate use of non-medical masks by the public. 8. Combine influenza vaccination with medical visit and / others vaccines (eg, pneumococcal vaccines) in same visit if possible so as to reduce health care encounters.	Looking at experience of Australia to adopt outdoor and drive through vaccine clinics.
Australia[27-33]	1. Aged care settings: everyone entering a residential aged care service (staff, visitors, health practitioners, volunteers and others) are required to be vaccinated since May 2020. Australian Government subsidised residential aged care providers are requested to provide free influenza vaccinations to staff and volunteers. 2. Healthcare workers are prioritised. 3. Increasing coverage for people older than 65 y.	1. The number of influenza vaccine doses increases by about 35% compared to the 2019 season. 2. More investment has been made to provide free influenza vaccines to vulnerable people through national influenza program. 3. Influenza coverage in the 2020 season is expected to be higher than the 2019 season as the number of vaccine doses that have been administered increases by 60% compared to the 2019 season and the vaccination continues to be offered. 4. Implement strategies to facilitate follow-up of patients requiring catch-up vaccination to avoid missing doses.	Immunisation providers, vaccinee and carers are required to comply with various measures to control the transmission of COVID-19, such as: 1. Maintaining physical distancing, by including additional administrative processes (eg, pre-booking immunisation services, separate staff administering vaccinations, monitoring of queueing), by including additional environmental measures (eg, display of signage, dedicated areas/rooms). 2. Including alternate models of providing service (eg, outdoor areas for vaccination). 3. Personal protective equipment should be made available for appropriate use in immunisation service settings. 4. Frequent environment cleaning practice for administration, clinical and patient areas (and between patient encounters). 5. Screen all attendees for suggestive symptoms of COVID-19 and assess those who have a possible exposure history (eg, travel and contacts with a COVID-19 patient). 6. Staff must not attend work if they are unwell. 7. Drive in immunisation clinics not routinely recommended due to safety concerns. Clear guidance is provided on location (parking area close to GP surgery), environment (clear signage, multiple parking bays free), clothing (loose fitting clothes to expose upper arm), monitoring post-vaccination (15 min).	Fever after vaccination should not be assumed to be due to receipt of an influenza vaccine and should be clinically assessed and tested for COVID-19.
New Zealand[34-36]	No change in the target groups, but influenza vaccination is prioritised to certain groups (details in next column).	Plan to increase coverage in several groups: 1. 75% for the population aged 65 y or older (about 63% have been vaccinated by early May 2020). 2. People under 65 y with medical conditions and pregnant women. 3. Healthcare workers (increasing the coverage to 80% or above).	Efforts are being made to expand access to influenza immunisation through other settings in addition to general practice and pharmacy, such as district health board staff health clinics and occupational health providers.	1. Influenza immunisation programme began earlier than usual for health care and other frontline workers (including emergency services, social services, police, defence and border control) and those at greatest risk of influenza. 2. The timeframe of influenza immunisation will be expanded.
UK [37-39]	1. All frontline health and social care workers will be urged to get their free vaccine, including frontline health and care workers in residential care and nursing homes, domiciliary care providers and the voluntary managed hospice sector (63%-70% of frontline health care workers received the influenza vaccine in 2016 to 2019 season). 2. Providing free vaccines for the first time for: (1) people aged 50 to 64 y; (2) people who are on the shielded patient list and members of their household; (3) children in secondary schools year seven.	According to a recent government press release, influenza immunisation programme will be expanded and more investment will be made. Some specific measures are considered to improve vaccination, such as using a proactive call and recall system to encourage influenza vaccination and continuing the school age influenza vaccination programme to invite all eligible children if possible.	No specific recommendations but some challenges in the coming season are anticipated. For example, vaccinees may be concerned about maintaining social distancing when being given the vaccine.	
Norway [40,41]	1. All personnel in health services with regular patient contacts should be offered free vaccine in the workplace. 2. An adjuvant influenza vaccine will be used to target residents older than 65 y in long-term care settings (eg, nursing homes) and people on the waiting list to these institutions and those older than 80 y receiving home care.	More influenza vaccines have been ordered (0.4 million more doses than last year).	1. Plan to include alternative vaccination sites. 2. Plan to implement infection control measures, including hand hygiene practice and infection control equipment. 3. Recommendation on infection control is available for different health institutions (such as general practitioner office and emergence department, nursing home, home care services).	
Germany [42,43]	Only people older than 60 y has been mentioned (same with the recommendation for previous seasons)	Vaccination providers should thoroughly check missed vaccinations.	1. Set up separate vaccination consultation hours and organise vaccination appointments. 2. Vaccination for people who have cold symptoms may have to be postponed. 3. Combine different vaccinations in one appointment.	
Greece [44]			1. Scheduled immunisation services to reduce crowding in the waiting areas. 2. Only one parent/carer is allowed. 3. Using face mask and hand hygiene. 4. For people who are self-isolated at home or receive care due to COVID-19 in hospitals, vaccination is recommended to be administered in 14 d after the disappearance of symptoms.	
The Netherlands [45]		300 000 extra doses of influenza vaccine have been secured this year (van der Hoek W 2020, personal communication, 4 September)	1. Vaccination at own practice or close by. 2. One-way systems at clinic reception and maintain physical distancing (1.5 m), through scheduled vaccination appointments, creating a separate entrance and exit, inviting vaccinees in phases, monitoring the queue and setting clear walking routes. 3. Discourage use of large-scale or drive through vaccination. 4. Combine influenza vaccination and pneumococcal vaccination if possible to reduce contacts. 5. Appropriate use of personal protective equipment. 6. For people with symptoms that indicate COVID-19, vaccinations are only carried out when the symptoms have disappeared. 7. For people with chronic diseases (eg, COPD), vaccination can be carried out with adequate protection for both patients and vaccinators.	
South Africa [46]	1. Influenza vaccination for all health care workers becomes mandatory and becomes the top priority funded influenza vaccination campaign in 2020. 2. People with hypertension, who were not in the priority groups of the 2018 recommendation, are included in 2020.	1. Influenza vaccination is highly recommended in the COVID-19 era. 2. Vaccination providers should thoroughly check missed vaccinations among children.	Immunization visits should continue uninterrupted in the COVID-19 era. Measures should be implemented to minimise the contact between individuals, such as maintaining physical distancing, scheduled appointments, and hand hygiene and cloth masks.	

We identified recommendations for the ongoing or upcoming influenza season in 13 countries, including five countries in the WHO regions of America, five in Europe, two in the Western Pacific region and one in the African region. These countries have adopted several novel immunisation strategies in addition to those in the regional and global recommendations (**Table 1**). For example, Canada, Germany and the Netherlands recommend combining influenza vaccination with other vaccines and / medical visits for each individual in one visit to reduce contacts [25,42,45]. Greece (and WHO regional office of America) allows only one carer to accompany the vaccine [44]. Canada, Greece and South Africa recommend vaccinees and carers use face masks appropriately [25,44,46]. Moreover, while Australia has set up outdoor and drive-through vaccination clinics, the Netherlands have recommended outdoor vaccination clinics but decided against drive-through clinics [27,45]. Canada will decide after looking at experience from Australia [25].

In addition to nonspecific recommendations on immunisation, some countries have made greater efforts to expand influenza vaccination programmes this year. For example, several Southern Hemisphere countries (eg, Australia, Chile and New Zealand) have increased their vaccination coverage substantially [20,28,34]. Norway, the Netherlands and UK have also planned to increase influenza vaccine coverage by making more investment to national influenza programmes and developing vaccination registry systems and catch-up programmes although the availability of substantially higher number doses of vaccine (compared to previous years) is a limiting factor [37,40,45]. With regard to priority groups, special attention has been paid to vaccinating health care workers and people in long term care facilities. For example, most of the countries included in our review have planned universal or nearly universal influenza vaccination for health care workers, or have made influenza vaccination for health care workers mandatory [26,35,38,40,46]. Australia has also requested all people entering long term care facilities to be vaccinated against influenza [29,30]. WHO (and the Regional office for the Western Pacific) also recommend that all residents and staff in long term care facilities be vaccinated against seasonal influenza [17,47]. Moreover, UK has recommended expanding the priority group list and additionally providing free influenza vaccine to all adults 50-64 years of age, people who are on the shielded patient list and members of their household and children in the first year of secondary school [37]. South Africa has additionally prioritised people with hypertension in 2020 [46].

## DISCUSSION

Several new strategies have been recommended or adopted at global, regional, and national levels to promote influenza vaccine uptake and control the spread of COVID-19 in immunisation settings.

Several countries included in this review have increased their investment in national influenza vaccination programmes or have planned to increase their investment to achieve a higher uptake in the upcoming influenza season. Influenza virus activity has been low in the past few months in the Southern Hemisphere due to the implementation of multiple stringent measures to decrease SARS CoV-2 transmission (eg, physical distancing, closure of schools, non-essential businesses, workplaces and other indoor settings), but the situation may be different for the Northern Hemisphere in the upcoming winter as it is unlikely to keep all the measures in place throughout the whole winter and the adherence with restrictions in the general population may decrease over time [1]. As the measures are gradually lifted, some settings (eg, schools and childcare) reopen and social activities increase, the transmission of influenza virus is expected to rise [48]. In such circumstances, a high influenza vaccine coverage is expected to be beneficial in the upcoming influenza season whist COVID-19 co-circulates and an effective COVID-19 vaccine does not yet exist [18,49,50]. This, however, can be challenging to achieve in many countries for several reasons. First, according to a recent survey, vaccination programmes in nearly 60 countries have been disrupted by the COVID-19 pandemic, making it difficult to maintain the provision of influenza vaccination services [16]. Second, due to the fear of COVID-19, people may be unwilling to leave home for vaccination [16]. To achieve a high influenza vaccine uptake, good communication strategies, vaccination record systems and catch-up campaigns are needed to promote the use of influenza vaccination services in the general population and identify missed vaccinations [15]. During the COVID-19 pandemic, there have been increasing concerns regarding vaccine hesitancy due to misinformation and public concerns about vaccine safety and its potential side effects [51,52]. Countries should build vaccine confidence by addressing public concerns regularly, enhancing community engagement, building public trust with health authorities, and helping health care workers build high-quality interactions with the community to address concerns [53]. On the other hand, the COVID-19 pandemic may have changed government and public perception about the importance of respiratory vaccines, leading to an increased demand for seasonal influenza vaccination for the upcoming season. Consistent with this, a recent study using Google trends data found a peak in worldwide interest in influenza and pneumococcal vaccines coinciding with the COVID-19 pandemic [54]. A sudden and large increase in demand may outpace the production capacity of seasonal influenza vaccines, which has not been augmented significantly in the most recent years [55]. It is of concern whether the manufacturing capacity is sufficient to match an increased demand for influenza vaccine at the global level. The availability of influenza vaccine is likely to be, as in the past, very limited in many countries, especially in low- and lower middle-income countries, given the uneven distribution of influenza vaccines across countries. According to a recent survey, the WHO African, Southeast Asia and Eastern Mediterranean regions altogether accounted for only 5% of the total number of influenza vaccine doses distributed globally in 2017 [56]. Additionally, countries may face challenges related to the need for increased vaccine storage and delivery capacity following an increase in the stock of influenza vaccine [19].

Prioritising influenza vaccination to the most vulnerable people can optimise the impact on population health. Healthcare workers are at increased risk of exposure to influenza virus [57,58]. Older adults, especially those in aged care settings, and people with medical conditions are at increased risk of developing severe disease and dying from influenza or COVID-19 infection [59-61] Vaccinating these groups is crucial in the upcoming season as it protects health care workers and prevents severe influenza infections, easing pressure on health care systems that have been already stressed by the COVID-19 pandemic [62-64]. Recent reviews have shown that influenza vaccination is safe, and not associated with increased risk of serious adverse events (eg, Guillain-Barré syndrome, respiratory function, cardiac arrest, and acute myocardial infarction), although may lead to a slightly increased risk of fever and local reactions such as tenderness, swelling and redness at the injection site [65-68]. There are no available data to suggest that the minor adverse effects of influenza vaccine lead to an increased risk of COVID-19 infections or severe outcomes. On the contrary, several studies found that people who were vaccinated against influenza were less likely to have severe COVID-19 compared to unvaccinated people [69].

Despite the substantial benefits of influenza vaccination, the delivery of influenza vaccination (and other vaccination) services can pose a challenge to the safety of vaccinators and the community in the context of COVID-19 because contacts between vaccinators, vaccinees and carers during vaccination visits may increase the risk of COVID-19 transmission. All the guidance identified in this review emphasizes the necessity to implement enhanced infection control measures in immunisation settings. Very specific measures have been adopted, which can be grouped into the following categories, including physical distancing measures, using alternative delivery models, locations and settings to provide vaccination (eg, outdoor areas, pharmacies and other health services), implementing hand hygiene practice and / using face masks, and ensuring the availability and appropriate use of personal protective equipment among vaccinators. The novel strategies (eg, combining influenza vaccination with other visits and setting up outdoor and drive through vaccination clinics) adopted by countries can help inform decision making in immunisation programmes in other countries. According to a recent modelling study, these measures, if implemented well, could be effective in reducing contacts between individuals and the risk of COVID-19 infection during vaccination visits [70]. However, in practice, implementation of these measures can be challenging and requires cooperative efforts. For example, countries need to develop detailed and specific instructions and provide clear guidance in different aspects and scenarios for vaccine providers and other health care workers at vaccination service delivery sites. Vaccine providers need to communicate with vaccinees and the community to ensure their understanding of and compliance with the infection control measures. Although we identified little information from resource-limited regions, implementing some of the infection control measures is also likely to be feasible and effective in these settings [70]. The availability of vaccine delivery infrastructures (eg, primary care services) and sustainable funding for vaccination programmes will facilitate national influenza vaccination policy formation in resource-limited regions. Strengthened vaccine delivery systems will also benefit the rapid introduction and up-scaling of new vaccines (eg, COVID-19 vaccine) in future [71].

This summary of guidance and strategies on influenza vaccination at global, regional and national level should support strategy development on influenza vaccination in the context of the COVID-19 pandemic.
